# The prevention and treatment of *Plasmodium vivax* malaria

**DOI:** 10.1371/journal.pmed.1003561

**Published:** 2021-04-23

**Authors:** Cindy S. Chu, Nicholas J. White

**Affiliations:** 1 Shoklo Malaria Research Unit-Mahidol Oxford Tropical Medicine Research Unit, Faculty of Tropical Medicine, Mahidol University, Mae Sot, Thailand; 2 Centre for Tropical Medicine and Global Health, Nuffield Department of Medicine, University of Oxford, Headington, Oxford, United Kingdom; 3 Mahidol Oxford Tropical Medicine Research Unit, Faculty of Tropical Medicine, Mahidol University, Bangkok, Thailand

## Abstract

Cindy S Chu and co-authors review options for diagnosis, safe and radical cure, and relapse prevention of Plasmodium Vivax.

**Summary points**

- The worldwide burden of *Plasmodium vivax* malaria has more than halved from an estimated 17.3 to 6.5 million cases between 2010 and 2019. This resulted from increased deployment of conventional malaria control measures (rapid diagnostic tests, effective antimalarial treatment, vector control) and significant global investment in malaria elimination. There is no generally available *P*. *vivax* vaccine, nor is there likely to be one in the near future.
- The latest-generation RDTs used for *P*. *vivax* diagnosis have sensitivities comparable to microscopy. Ultrasensitive PCR methods which can detect parasite densities as low as 28/ml have revealed a much higher prevalence of asymptomatic *P*. *vivax* infection in malaria endemic regions than previously estimated.
- Chloroquine remains an effective schizonticide for vivax malaria, except in Indonesia, Sabah and Papua New Guinea where there is high-level chloroquine resistance. Artemisinin combination therapies are effective alternative schizonticides that unify the treatment of all malarias. Relapses contribute significantly to the burden of *P*. *vivax* infections. Prevention of relapse requires “radical cure” with an 8-aminoquinoline (primaquine daily for 7 to 14 days, or single-dose tafenoquine). These drugs cause oxidant haemolysis in G6PD deficiency, so safe use requires G6PD testing. Overall, the risks of primaquine haemolysis have been overemphasised and the benefits of relapse prevention underappreciated.
- Qualitative screening tests for G6PD deficiency (detecting <30% normal enzyme activity) are adequate for screening before giving primaquine for radical cure, but a quantitative point of care G6PD test to detect <70% normal enzyme activity is needed for tafenoquine.
- Radical curative efficacy depends on the total 8-aminoquinoline dose given; higher primaquine doses (7 mg base/kg rather than 3.5 mg base/kg) are required in parts of Southeast Asia and Oceania. The currently recommended dose of tafenoquine (300 mg) is sub-optimal. In low transmission settings, elimination of vivax malaria is possible with current tools.

## Introduction

*Plasmodium vivax* is more geographically dispersed than *Plasmodium falciparum*, with transmission occurring over a wider range of temperatures than for *P*. *falciparum* [1], and at latitudes as far as 64° North [2]. *P*. *vivax* is the predominant cause of human malaria in Asia and the Asia-Pacific regions where, with large populations and a declining incidence of *P*. *falciparum* infections, over 80% of the global *P*. *vivax* burden occurs [3]. *P*. *vivax* is also prevalent in the horn of Africa, Madagascar, and parts of Central and South America, but it has been eradicated from Europe, Russia, North America, and most of the Middle East [2,4].

Clinical management of *P*. *vivax* malaria relies on clinical suspicion, a reliable blood diagnostic, and access to efficacious effective schizonticidal (blood stage) and hypnozoiticidal (radical cure) drug regimens. Currently, *P*. *vivax* malaria is treated with chloroquine or artemisinin combination therapy (ACT) for the blood stage infection. While chloroquine has been standard treatment for vivax malaria for some 70 years, the emergence and global spread of chloroquine resistance in *P*. *falciparum* has meant that different treatments are now required for *P*. *falciparum* and the other human malarias. Use of ACTs (except artesunate-sulfadoxine-pyrimethamine) allows again a unified treatment for all malaria, and it provides a safety net in case of misdiagnosis or an unidentified mixed infection with *P*. *falciparum*. Despite declining susceptibility, chloroquine is still effective for *P*. *vivax* infection in most of the world, except for Indonesia, Sabah and Oceania where resistance is high grade [5,6]. Chloroquine is substantially less expensive than ACTs, although a unified treatment provides significant operational cost-savings.

Historically, *P*. *vivax* has been considered benign, although severe infections may sometimes occur [7–10]. Concomitant illness or chronic disease may contribute to the severity of *P*. *vivax* infection [11,12]. The management of severely ill patients with *P*. *vivax* malaria is similar to that of *P*. *falciparum* malaria [13]. *P. vivax* malaria in pregnancy is associated with miscarriage and low birth weight [14,15].

The great challenge for *P*. *vivax* malaria treatment is how to prevent relapses. These result from dormant liver stage parasites (hypnozoites) that activate weeks or months after the primary infection to cause a recurrent *P*. *vivax* infection (relapse). The intervals to and frequency of symptomatic relapses vary geographically and depend also on age and the intensity of transmission [16–18]. Frequent *P*. *vivax* infections are a major cause of morbidity in vivax malaria, particularly in young children in whom repeated relapse may cause severe anaemia, malnutrition, and growth delay [19, 20]. Thus, an important goal of treatment is to prevent relapse. This requires radical curative treatment with an 8-aminoquinoline in addition to treatment of the blood stage infection. The 8-aminoquinoline drugs cause haemolysis in patients with glucose-6-phosphate dehydrogenase deficiency (G6PDd), and this risk of dangerous iatrogenic haemolysis has limited their use substantially. In general, the harm caused by frequent relapse, particularly in young children, has been under appreciated, whereas the risks of haemolysis have been overemphasised, with a net result that primaquine radical cure has been underused.

### Diagnosis of *Plasmodium vivax* malaria

#### Microscopy and rapid diagnostic tests

For over a century, the gold standard for diagnosing *P*. *vivax* malaria has been the microscopy observation of asexual parasites on a thick or thin blood film. Accurate microscopy requires trained laboratory staff supported with adequate laboratory supplies and well-maintained microscopes, which are not always available in resource-limited settings. The limit of detection for expert microscopy is about 50 parasites/uL which coincides approximately with the pyrogenic density [21]. The diagnostic sensitivity depends on the slide quality and the experience of the microscopist. Thus under field conditions, microscopy may miss some symptomatic cases because of the low parasite densities in *P*. *vivax* infections, but overall, it is suitably sensitive and specific for clinical diagnosis. Rapid diagnostic tests (RDTs) have been developed which detect antigens common to all malarias (e.g., aldolase) or those specific to *P*. *vivax* (e.g., *Pv*LDH). First-generation RDTs had lower sensitivities for *P*. *vivax* parasitaemia [22] and could give false positive results [23] with high-density *P*. *falciparum* infections. The sensitivity and specificity of *P*. *vivax* RDTs have both been improved significantly. From 2008 to 2018, the proportion of *P*. *vivax*, *P*. *falciparum*, and pan-malaria RDTs meeting the current WHO performance threshold for procurement increased from approximately 20% to 90% [24]. During that decade, over 2.3 billion RDTs have been sold [25]. Access to reliable point-of-care diagnostic tools has allowed for early diagnosis and improved tremendously the ability to treat confirmed rather than suspected *P*. *vivax* infections.

#### Polymerase chain reaction (PCR) detection and genotyping

PCR allows low densities of malaria parasites to be detected reliably in blood samples. For low-volume blood samples such as filter paper blood spots, lower limits of reliable detection are typically in the range of 1 to 10 parasites/uL. In recent years, high-volume ultrasensitive PCR methods (uPCR) have been developed which amplify a larger amount of DNA, and these provide lower limits of detection around 28 parasites per mL (based on genus) with a second species-specific probe based on the 18S RNA gene [26]. These ultrasensitive methods allow detection of the majority of all infected people in endemic areas [27]. Most of these malaria-infected people are asymptomatic although, as parasite densities fluctuate over time, transmissible densities of gametocytes occur intermittently, and they are therefore a source of transmission [28]. PCR identification of infection is therefore an important component of epidemiological or clinical research assessment for submicroscopic recurrences, but it should not be used in disease diagnosis and management as it is too sensitive. That is, it will detect asymptomatic individuals who often have another reason for their febrile illness (reduced specificity).

Molecular genotyping is not needed for clinical management, although it is useful in clinical trials where, together with time-to-event information, it can help distinguish relapse from reinfection [29]. The interpretation of parasite genotyping is not as straightforward as it is for *P*. *falciparum* infections as relapses can be with genotypes similar or identical to the initial infection, or they can be with completely different genotypes resulting from heterologous hypnozoite activation [30, 31]. Nevertheless, combining genotype comparison with time-to-event information does allow probabilistic differentiation between relapses and reinfections.

### Prevention

#### Insecticide-treated nets

Insecticide-treated nets (ITNs) are the mainstay of malaria control in most of the malaria-endemic world. Between 2017 and 2019, over 25,000,000 ITNs were distributed annually in malaria-endemic regions outside sub-Saharan Africa [4]. The effectiveness of ITNs depends on many factors which include distribution, coverage, adherence, maintenance, vector biting patterns, and levels of insecticide resistance [32]. Unsurprisingly ITNs are less effective if the anopheline vectors bite when people are not under or near their bed nets [33,34]. Most of the main vectors in SE Asia exhibit exophilic crepuscular biting patterns which decrease the effectiveness of ITNs in preventing malaria. In Papua New Guinea, anopheline vectors changed their behaviour to bite earlier after the introduction of bed nets [35]. Moreover, ITNs have no direct impact against relapse, which is often the main cause of vivax malaria (and once relapse proportions exceed 50%, relapses become the main cause of *P*. *vivax* infections). Despite these limitations, ITNs do provide partial benefit and are an adjunct method to prevent primary *P*. *vivax* infections and interrupt transmission [36]. Long-lasting nets (LLINs), in which the insecticide persists for the natural life of the net, are a substantial advance. There is rising concern about increasing pyrethroid resistance. Progress against pyrethroid resistance has been made in the forms of a new pyrrole insecticide, chlorfenapyr [37,38], and a chemical synergist which prevents insects from detoxifying pyrethroids, piperonyl butoxide (PBO) [39].

#### Insecticide use

The use of insecticides for malaria prevention focuses mainly on indoor residual spraying (IRS) and long-lasting ITNs, although there are a variety of other approaches. Organochlorides, organophosphates, carbamates, and pyrethroids are recommended by WHO for IRS. From 2000 to 2009, over 3,200 metric tonnes of insecticide were used against malaria vectors in *P*. *vivax* endemic regions [40]. Widespread insecticide use for agricultural treatments and large-scale malaria control programmes create intense selection pressure on vector populations to develop insecticide resistance [41,42]. Resistance to organochlorides and pyrethroids has been detected in the Americas, Asia, SE Asia, and the southern Pacific [42,43] where *P*. *vivax* is endemic, though even within a single country, resistance patterns may vary [44]. The full extent of insecticide resistance is not well characterised because routine monitoring in anopheline vector populations is not performed in all countries.

#### Vaccines

There is no generally available *P*. *vivax* vaccine, and none is on the near horizon. In contrast to *P*. *falciparum*, where hundreds of clinical trials and nearly 20 preclinical trials are registered, only 1 clinical trial and 2 preclinical trials are registered for *P*. *vivax* [45]. Potential vaccine strategies for *P*. *vivax* are irradiated sporozoites [46] and the blood stage antigens *P*. *vivax* Duffy binding protein (PvDBP), *P*. *vivax* merozoite surface proteins (PvMSP1, PvMSP3α, PvMSP9), *P*. *vivax* apical membrane antigen (PvAMA1), and the liver stage antigen *P*. *vivax* circumsporozoite protein (PvCSP) [47]. Transmission-blocking antigens are also potential targets. Phase I human trials have been conducted with PvCSP [48] and PvDBP [49] and asexual stage antigen Pv25 [50,51]. Currently, a single Phase I *P*. *vivax* vaccine trial (of a viral vectored PvDBP vaccine) is listed on the global WHO malaria clinical vaccine projects “Rainbow tables” (Trial NCT01816113) [45]. Vaccine development for *P*. *vivax* faces different challenges than with *P*. *falciparum*. *P*. *vivax* has greater genetic diversity [52] and cannot be maintained reliably in continuous cultures in vitro.

#### Chemoprophylaxis

Chemoprophylaxis is recommended to protect travellers from nonendemic areas entering a malaria-endemic area. Recommendations are largely focused on *P*. *falciparum*, the more dangerous malaria parasite, both to prevent severe disease, and also because nearly all the drugs which work against *P*. *falciparum* (either in pre-erythrocytic or “causal” prophylaxis, or in blood stage “suppressive” prophylaxis) are also effective against *P*. *vivax*. Intermittent presumptive treatment for infants and pregnant women (IPTi and IPTp) and seasonal malaria chemoprevention (SMC) are interventions which aim to prevent *P*. *falciparum* blood stage infections in populations that live in areas of moderate to high *P*. *falciparum* transmission. These are typically areas where *P*. *vivax* is either uncommon or absent. The only suppressive chemoprophylactic strategy specifically targeting *P*. *vivax* for endemic populations is a weekly chloroquine dose of 5 mg base/kg (300 mg base adult dose) given during pregnancy and lactation [53] as 8-aminoquinolines are contraindicated in this population (Table 1). This is seldom used, probably because of insufficient cost-effectiveness where incidence is low. But the cost-effectiveness balance does favour chloroquine prophylaxis to suppress relapse in pregnancy in women following an incident tropical (frequent relapse) *P*. *vivax* infection—and then it should be given. The role of IPTp with dihydroartemisinin-piperaquine in parts of Indonesia and Papua New Guinea, where transmission and resistance to chloroquine are both high, is under investigation with promising results in clinical trials [54].

**Table 1:** Chemoprophylactic regimens against *Plasmodium vivax* infection.

Indication and treatment	Contraindications^a^	Dose	When to start	Treatment duration
**Suppressive chemoprophylaxis in pregnancy**				
Chloroquine	Severe renal or hepatic disease, can exacerbate psoriasis^a^	300 mg base weekly	First antenatal visit or 3 weeks after treatment of vivax malaria	Continue until delivery
**Suppressive chemoprophylaxis in travellers**				
Any antimalarial regimen that is used for *P*. *falciparum* chemoprophylaxis (doxycycline or mefloquine)	Doxycycline: Myasthenia gravis, children <8 years, second or third trimester pregnancy Mefloquine: Epilepsy, history of or current psychiatric disease, severe hepatic disease	Doxycycline 100 mg daily Mefloquine 250 mg base weekly	1 day before travel 4 weeks before travel	Continue for 4 weeks after return to nonendemic area
**Causal prophylaxis in travellers**				
Primaquine	G6PD deficiency, pregnancy, mothers breast feeding infants (<6 months)^b^ or those who are G6PD deficient, and infants <6 months	30 mg base daily with food	1 day before travel	Continue until 7 days after return to nonendemic area
Tafenoquine^c^^,^^d^	G6PD deficiency (quantitative testing showing >70% normal activity required), pregnancy, mothers breast feeding infants who are G6PD deficient, history of psychotic disorder or current psychiatric symptoms	Loading dose of 200 mg base daily for 3 days, then 200 mg weekly with food (maximum 6 months)	1 day before travel	
Atovaquone-proguanil	Pregnancy, severe renal disease (GFR <30 mL/min)	250 mg/100 mg (1 tablet) daily with food	1 day before travel	
**Presumptive antirelapse therapy in travellers (PART)–necessary after suppressive chemoprophylaxis**				
Primaquine	Same as for the causal prophylaxis indication	0.25–0.50 mg base/kg/day with food	7 days after return to the nonendemic area	14 days
Tafenoquine^d^ (currently recommended only in combination with chloroquine treatment or chemoprophylaxis)^c^	Same as for the causal prophylaxis indication, except that history of psychiatric disorder or current psychiatric symptoms are not a contraindication	300 mg base with food	7 days after return to the nonendemic area	Single dose

a Contraindications listed are in addition to allergy and apply only to the prophylaxis and not treatment indication.

b Minimal primaquine was excreted into mature breast milk in a study of breast feeding infants >28 days old [57].

c Tafenoquine product labelling has been changed so that only chloroquine may be used as the partner schizonticide [58,59]. Paediatric dosing recommendations are anticipated (TEACH trial) [60].

d The US Centers for Disease Control and Prevention (CDC) malaria guidance extends the use of tafenoquine for radical cure to PART [61].

GFR, glomerular filtration rate; G6PDd, glucose-6-phosphate dehydrogenase deficiency; PART, presumptive antirelapse therapy.

As countries eliminate malaria, the infections they encounter will increasingly be in travellers arriving from endemic areas. Suppressive chemoprophylaxis with drugs effective against *P*. *falciparum* has been the primary approach for malaria prevention (e.g., the same regimens apply to *P*. *vivax*), although they do not prevent the establishment of hypnozoites. The 8-aminoquinolines (primaquine and tafenoquine) have good activities against the pre-erythrocytic stages of all malarias, as well as blood stage activities against *P*. *vivax*, and so can be used for chemoprophylaxis. These drugs prevent later relapses as they kill hypnozoites (whereas IPTi or SMC with schizonticides only delay multiplication of the blood stage). Atovaquone-proguanil is also considered to provide highly effective causal prophylaxis against *P*. *vivax* but it does not prevent relapses. Recent observations in travellers showed a similar rate of *P*. *vivax* infections (40% to 50%) up to 1 year after returning to a nonendemic area following either atovaquone-proguanil, mefloquine, or doxycycline prophylaxis. This compares with a rate of only 1% to 2% in travellers who had received primaquine [55]. This suggests that while atovaquone is active against the developing hypnozoite, it is not active against the metabolically inert hypnozoite more than 3 days postinoculation [56]. The drugs, which act primarily on the liver stage (pre-erythrocytic) infection (i.e., causal prophylaxis), have the substantial advantage that they can be stopped within days of leaving the endemic area, whereas the blood stage suppressive drugs (mefloquine, tetracyclines, chloroquine) need to be continued for 1 month after leaving to capture late emerging blood stage infections. To ensure that relapses from *P*. *vivax* and *Plasmodium ovale* are prevented presumptive antirelapse therapy (PART) with a radical curative dose of 8-aminoquinolines (primaquine and tafenoquine) may be given following departure from an endemic area to persons receiving suppressive chemoprophylaxis (Table 1). The timing of the PART dose is important as it should be started while blood schizonticide concentrations remain above the mean inhibitory concentration. Thus, PART can be started within the final 2 weeks of doxycycline and mefloquine, and the final week of atovaquone-proguanil suppressive chemoprophylactic regimens.

### Treatment of acute *Plasmodium vivax* malaria

#### Blood stage treatment

Since 1947, chloroquine has been the treatment of choice for blood stage infections with *P*. *vivax*. The standard treatment regimen is 25 mg of base equivalent/kg divided over 3 days and given either as 10 mg/kg initially followed either by 10 mg/kg at 24 hours and 5 mg/kg at 48 hours, or by 3 doses of 5 mg/kg 12 hourly [53]. Various other ways of giving this 25 mg/kg treatment dose have been recommended, and chloroquine may be administered safely over 36 hours provided that individual doses do not exceed 10 mg/kg. Although lower doses are effective, there is no reason to depart from this standard regimen in most regions. Absorption of chloroquine by mouth is reliable even when patients are prostrate [62], and there is seldom need for parenteral treatment. Indeed, parenteral formulations of chloroquine are no longer generally available, so those patients who do require initial parenteral treatment for severe *P*. *vivax* malaria should be given intravenous or intramuscular artesunate until they can take oral medication reliably [13]. ACTs are also highly effective (except for artesunate-sulfadoxine-pyrimethamine) and provide a slightly more rapid clinical and parasitological response than chloroquine [63–67]. The post-treatment suppression of early relapses depends on the elimination kinetics of the ACT partner drug—mefloquine, piperaquine, pyronaridine, and amodiaquine ACTs provide approximately similar durations of suppression compared with chloroquine, whereas artemether-lumefantrine provides a significantly shorter period of suppression [68]. Both chloroquine and ACTs are generally well tolerated antimalarial treatments. The weight-adjusted schizonticidal treatment regimens are similar in pregnant and lactating women [54]. Paediatric formulations are available for artemether-lumefantrine, artesunate-amodiaquine, dihydroartemisinin-piperaquine, and pyronaridine-artesunate, and the doses are similar to those recommended for falciparum malaria. Child-friendly formulations and smaller-dose tablets are needed for primaquine.

Primaquine and tafenoquine both have significant blood stage activities but as the therapeutic response is significantly slower than with chloroquine or ACTs, they should not be used as monotherapies for blood stage infections [69,70]. Nevertheless, the asexual stage activity of 8-aminoquinolines does contribute significantly to the treatment efficacy and prevents resistance to standard schizonticides by providing a combination treatment for the blood stage infection. *P*. *vivax* gametocytes are considered as sensitive as the asexual stages to antimalarial drugs [71] so, unlike *P*. *falciparum*, specific gametocytocidal treatment is not necessary.

High fevers can be managed with standard doses of paracetamol, and vomiting can be treated with antiemetics. If patients show signs of severe vivax malaria, then the management is exactly the same as for severe falciparum malaria [13]. Uncomplicated mixed infections with *P*. *falciparum* and *P*. *vivax* should be treated with an ACT and a radical curative regimen [53].

#### Antimalarial drug resistance

**Fig 1:**
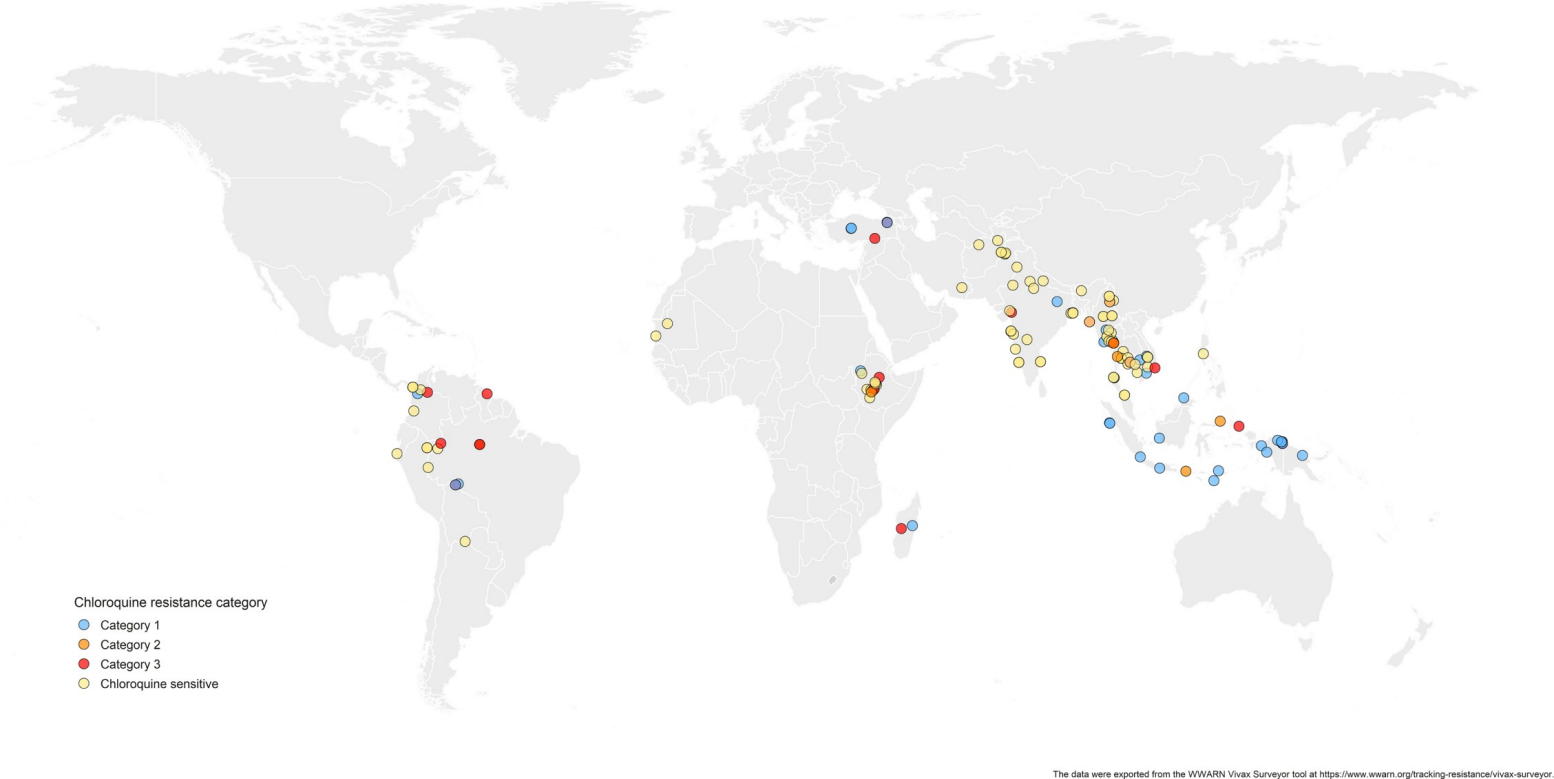
Levels of chloroquine resistance against *Plasmodium vivax* from 1985–2019. The data were exported from the WWARN Vivax Surveyor tool at https://www.wwarn.org/tracking-resistance/vivax-surveyor. Evidence for chloroquine resistance comes from *P*. *vivax* clinical trials published from 1985–2019. Some trials are from different years in the same area; this figure does not specify the change in chloroquine resistance pattern over time. **CQS**: Chloroquine sensitive, **Category 1:** very suggestive of resistance, **Category 2:** suggestive of resistance, **Category 3**: potential evidence of resistance.

Chloroquine resistance in *P*. *vivax* has emerged more slowly than for *P*. *falciparum*. The mechanism of resistance and its molecular basis have proved elusive. Evidence of chloroquine resistance was detected first in a traveller returning from Papua New Guinea over 30 years ago [5,6]. Chloroquine resistance has been defined as a patent *P*. *vivax* infection in the presence of chloroquine + desethychloroquine levels >100 ng/mL in whole blood or >10 ng/mL in plasma [72]. Since then, chloroquine resistance has risen to high levels in Indonesia and Oceania (Fig 1) and so ACTs are used instead of chloroquine in this region. Dihydroartemisinin-piperaquine, artesunate-mefloquine, artemether-lumefantrine, and artesunate-pyronaridine are all very effective treatments of vivax malaria [62–65]. While amodiaquine is also more effective than chloroquine against resistant *P*. *vivax*, it is not as well tolerated as dihydroartemisinin-piperaquine, and resistance in *P*. *falciparum* is widespread in *P*. *vivax*-endemic regions. Amodiaquine-containing regimens are not therefore recommended for chloroquine-resistant *P*. *vivax* infections [73–74]. Despite evidence for slowly rising chloroquine resistance in *P*. *vivax*, chloroquine still remains effective throughout most malaria-endemic areas [65,75–79]. As the concomitant use of primaquine (for radical cure) provides significant independent asexual stage activity, it may mask low-level chloroquine resistance [80–82]. The lack of a molecular marker of chloroquine resistance and the limited availability of in vitro testing in vivax malaria [83] means that the epidemiology of chloroquine resistance is not well described. Antifol resistance occurs readily in *P*. *vivax* through mutations in *Pvdhfr* which are readily identified. Antifol resistance is geographically widespread [84,85]. *P*. *vivax* is also intrinsically relatively resistant to sulphonamides. This is why the artesunate-sulfadoxine-pyrimethamine ACT should not be used for *P*. *vivax* treatment.

#### *Plasmodium vivax* relapse

**Fig 2:**
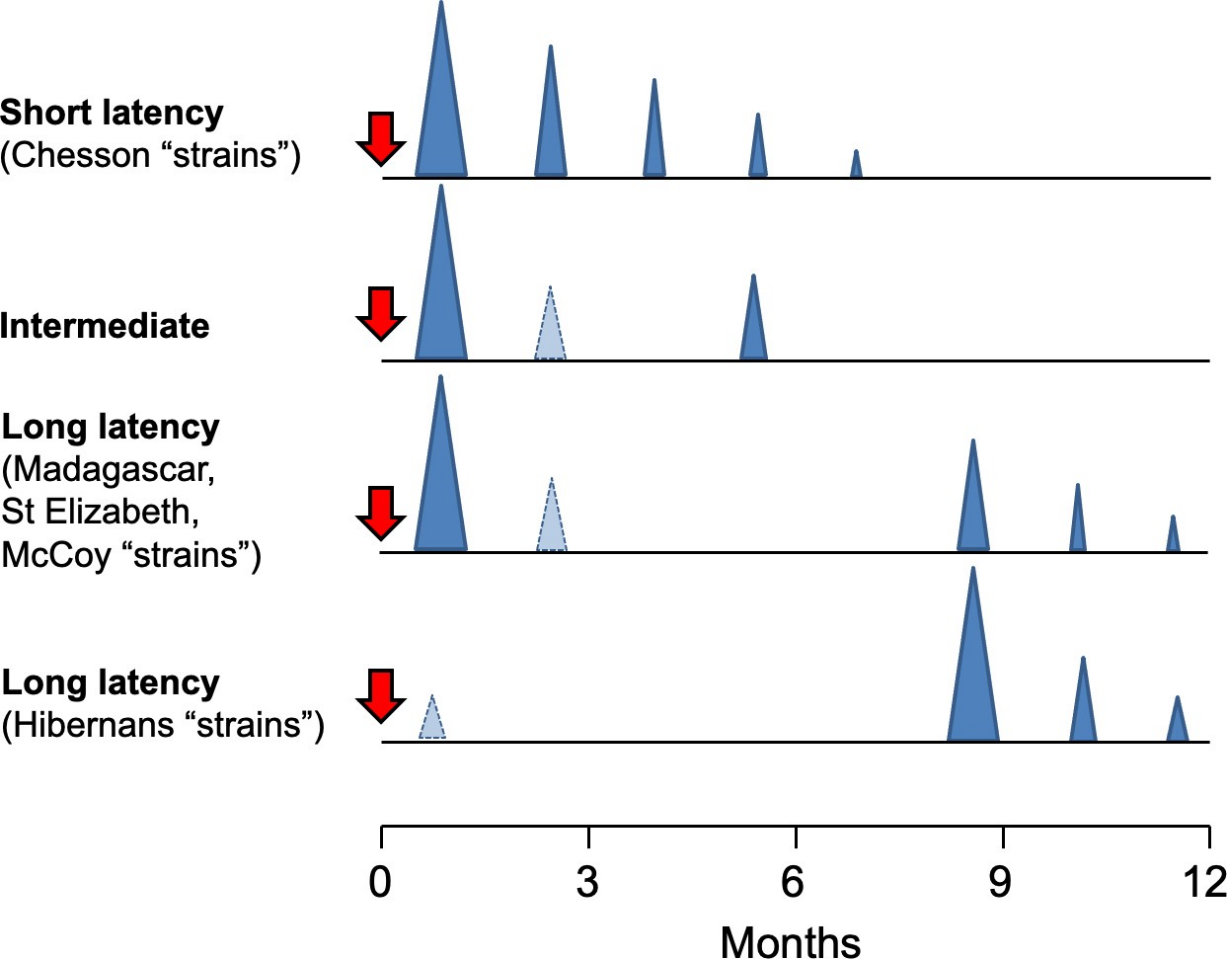
*Plasmodium vivax* relapse patterns. The temporal patterns of *P*. *vivax* relapse in different “strains.” The red arrow indicates the infective mosquito bite which leads to the primary infection. The blue triangles represent patent *P*. *vivax* infections; the largest triangle is the primary infection. The proportions of successive relapses decline, and there is an increasing probability that the relapses are oligosymptomatic or asymptomatic. The translucent blue triangles are *P*. *vivax* infections which may sometimes occur. The short latency frequent relapse pattern (typified by the “Chesson strain”) is prevalent across tropical areas. The intermediate phenotype may occur in South Asia. The long latency phenotype (typified by the Madagascar, St Elizabeth, and McCoy strains) is found in Central America, North Africa, and central Asia, while the long latency “hibernans” phenotype, which was prevalent in Northern Europe and Russia, is still found in North Korea.

Relapses of *P*. *vivax* and *P*. *ovale* are the recurrent blood stage infections which originate from hypnozoites in the liver. Relapses occur with periodic regularity (Fig 2); short latency phenotypes prevalent in tropical climes relapse at approximately 3- to 4-week intervals. These intervals are prolonged by slowly eliminated antimalarial drugs. Longer latency phenotypes may relapse 8 to 9 months after a primary infection [86]. The proportion of infections which relapse varies between 20% and 100% depending on location and transmission intensity.

The multiplication of the blood stage infection of early relapses is suppressed by slowly eliminated antimalarials (e.g., chloroquine) prescribed for the previous clinical *P*. *vivax* episode. As a consequence, in tropical areas, relapses become patent 3–6 weeks after artemether-lumefantrine and 5–7 weeks after chloroquine, whereas they occur around 3 weeks after artesunate or quinine treatment [76].

### Antirelapse (radical cure) treatment

#### Primaquine

For over 60 years, the mainstay of antirelapse treatment has been a 14-day primaquine regimen. Divergent policies and practices, concerns over haemolysis, and the unavailability of G6PD testing have contributed to low rates of primaquine uptake for radical cure in malaria-endemic countries [87].

The radical curative efficacy of primaquine depends on the total dose given. There have been relatively few randomised trials in which the radical curative efficacy of primaquine has been assessed with a necessary ≥4-month follow-up (1 year is preferable). In South America, 7-day regimens are used. The radical curative efficacy of the 7-day low-dose (total 3.5 mg base/kg) primaquine regimen was approximately 80% [88–92]. In SE Asia and Oceania, higher doses are needed for maximum efficacy [93]. The most commonly recommended higher dose is a total of 7 mg base/kg given over 14 days (adult dose 30 mg/day), although most countries still recommend the lower dose (3.5 mg base/kg total dose; adult dose 15 mg/day for 14 days or 30 mg/day for 7 days). Potentially poor adherence to the 14-day treatment reduces the effectiveness of radical cure. Recent studies show that shortening the course of the higher-dose treatment to 7 days (adult dose 60 mg/day) is efficacious and safe in G6PD normal patients, although significant haemolysis may occur in heterozygote females with intermediate G6PD activity who have tested normal with standard qualitative G6PD screening tests [94,95] (see below). G6PD quantitative tests may be needed for this short-course high-dose treatment. For patients who test as G6PD deficient, a weekly primaquine dose (0.75 mg base/kg/dose) given for 8 weeks has been recommended, although the safety of this dose has only been assessed in a few populations [53,96–98].

In early clinical trials, it was observed that chloroquine potentiated the radical curative efficacy of primaquine [99]. The mechanism underlying this relationship and its clinical significance remain unclear. Radical curative efficacy is similar when primaquine is paired with chloroquine or an ACT, and radical cure rates over 95% can be achieved after supervised dosing [29,94,95]. Primaquine requires metabolism to its bioactive metabolites by the liver isoenzyme CYP2D6 [100,101]. Primaquine failure may therefore occur in patients with CYP2D6 mutations associated with a poor or intermediate metaboliser phenotype. Overall, adherence likely plays a greater role in the failure of primaquine to prevent relapse. Resistance to primaquine has been reported, but there is no conclusive evidence of acquired resistance in liver stages.

Primaquine is contraindicated in pregnancy, is not recommended in infants <6 months, and has also been withheld from lactating women, although a recent study shows that the dose ingested by the breastfeeding infant is very small [57] and therefore very unlikely to exert any biological effect. This restriction may well be lifted. Primaquine is generally well tolerated. It may cause abdominal pain if larger doses (over 0.5 mg/kg) are taken on an empty stomach. This is lessened if given with food. The main adverse effect of the 8-aminoquinolines is dose-dependent oxidant haemolysis in G6PD deficiency. Primaquine-induced methaemoglobinaemia is usually mild, but it can be severe in NADH methaemoglobin reductase deficiency.

#### Haemolysis in G6PD deficiency

G6PD deficiency is the most common human enzymopathy. Gene frequencies average 8% to 10% across much of the tropical world [102]. The risk of haemolysis in G6PD deficiency is the main obstacle to the use of 8-aminoquinolines for radical cure. The enzyme deficiency is X-linked so males are either normal or deficient (hemizygotes), whereas women can be either normal, or fully (homozygotes) or partially deficient (heterozygotes). On average, at a population level, female heterozygotes have half their red cells which are normal and half which are G6PD deficient but, because of random X-inactivation (Lyonisation), these proportions vary substantially between individuals, thus causing wide variations in the degree of enzyme deficiency among female heterozygotes. Both primaquine and tafenoquine cause predictable dose-related haemolysis in G6PD-deficient individuals [103,104]. Haemolysis disproportionately affects older red blood cells because G6PD activity declines as red blood cells age [105]. There are approximately 200 different polymorphic G6PD deficiency genotypes [106]. The majority confer reduced enzyme activity, although the degree of deficiency varies substantially among the different genotypes. The extent of haemolysis depends on the dosage of drug and the genotype. In Africa, the less severe A-genotype is prevalent, whereas in Asia, the common genotypes confer greater enzyme deficiency. The most commonly used screen for G6PD deficiency is the qualitative G6PD fluorescent spot test (FST), which detects the naturally fluorescent NADPH. The FST detects <30% of normal G6PD activity reliably (deficient blood does not fluoresce) so it identifies the male hemizygotes, female homozygotes, and those heterozygotes with G6PD activity near the 30% threshold. The FST may not be feasible at lower-level healthcare settings as it requires electricity, trained laboratory staff, and quality control. New qualitative lateral flow G6PD RDTs are available. These can be performed where the FST cannot. These RDTs are an important support tool for diagnosing G6PD deficiency, and the safe prescription of primaquine as *P*. *vivax* radical cure is scaled up. Patients who have a test result indicating G6PD deficiency should not receive the standard primaquine regimen. Instead, in areas where the less severe G6PD variants (e.g., G6PD A-) are prevalent, they should receive a once weekly 0.75 mg base/kg dose for 8 weeks [53,96]. The safety of the weekly regimen has not been established in patients with G6PD variants that are severe (e.g., G6PD Mediterranean). However, a threshold for phenotypic severity has not been defined. Even with FST or RDT screening, there may still be significant haemolysis in G6PD heterozygote females who have slightly more than 30% of G6PD normal erythrocytes. Such females would be considered as G6PD normal and potentially given primaquine. Unfortunately, in many regions, G6PD testing is unavailable so the practitioner treating vivax malaria is faced with a dilemma—to give radical cure “at risk,” or to err on the side of safety and not give it. In this difficult choice, the substantial morbidity of recurrent *P*. *vivax* malaria, particularly in young children who may be ill every second month, has often been underappreciated [107]. Importantly, in gauging the risk, it is important to note that G6PD deficiency protects against *P*. *vivax* malaria, so at a population level, the risk of haemolysis in patients is lower than would be predicted from prevalence surveys in healthy individuals [108]. In a recent study in Afghanistan of Pashtun patients (in whom the main G6PD variant is the “severe” Mediterranean variant), the prevalence of G6PD deficiency in men (i.e., hemizygotes) presenting with vivax malaria was 4 times lower than in the healthy population [109]. The result of these various uncertainties has been a diverse array of National treatment recommendations and practices for radical cure. Many countries do recommend giving primaquine “at risk,” yet despite over 60 years of widespread use, the total number of documented fatalities from haemolysis is only fifteen [110,111]. The likely reason for this low number is that primaquine can be stopped as soon as there are symptoms or clinical evidence of substantial acute haemolysis (e.g., lassitude, exertional dyspnoea, haemoglobinuria, or abdominal discomfort) and, because primaquine and its oxidant metabolites are rapidly eliminated, the anaemia is limited. This probably explains why mass treatments with primaquine without G6PD testing, even in areas where G6PD Mediterranean was the main deficiency genotype, were not apparently associated with serious toxicity [110].

#### Tafenoquine

Tafenoquine is a slowly eliminated 8-aminoquinoline (terminal elimination half-life of approximately 12 days) which allows single-dose treatment. As with primaquine, tafenoquine is generally well tolerated and abdominal discomfort is lessened if given with food. During the long development of tafenoquine, concerns were raised about the potential for vortex keratopathy, electrocardiograph QT prolongation, and psychiatric reactions. The clinical evidence to date has largely resolved these issues [112–115] although, in the product labelling, tafenoquine for causal prophylaxis is considered contraindicated in persons with psychiatric symptoms. Being a single-dose treatment, tafenoquine addresses a major limitation of the 7- or 14-day primaquine regimen, the potential for poor adherence. However, this advantage is at the expense of a substantial risk of haemolytic toxicity in G6PD deficiency as, once tafenoquine is taken, it cannot be “stopped” if there is drug-induced haemolysis. A meta-analysis of the multicountry phase 3 studies showed that tafenoquine had a 6-month radical curative efficacy similar to low-dose primaquine (0.25 mg/kg/day for 14 days) in South America (67% versus 66%, respectively) and the horn of Africa (75% versus 80%, respectively), but the efficacy of the currently recommended 300 mg dose was significantly inferior in the SE Asian sites (74% versus 93%, respectively) [116,117]. These are disappointing radical curative efficacies and suggest that higher tafenoquine doses are probably needed in SE Asia and Oceania where *P*. *vivax* relapse rates are high, and also in South America where both radical cure regimens performed poorly [118]. Tafenoquine efficacy did not appear to be affected significantly by CYP2D6 polymorphisms in the Phase III trials [119], but more data are needed to confirm these early findings.

As described, tafenoquine’s advantage in being slowly eliminated (terminal half-life approximately 12 days), and thus effective in a single dose, is also its Achilles heel. If given to an individual who is G6PD deficient, the drug exposure and resulting haemolysis will persist until all the susceptible erythrocytes have been destroyed. Some female heterozygotes, who would be undetected by the standard screens, could still theoretically lose up to 70% of their red cells. This is why the higher G6PD activity threshold (>70% G6PD activity) has been chosen for tafenoquine eligibility. The gold standard method for G6PD quantitation is spectrophotometry requiring relatively expensive and sophisticated laboratory techniques, so it is not feasible in nearly all healthcare settings. Thus, for safe use of tafenoquine, new quantitative point-of-care G6PD tests have been developed. Studies to validate G6PD quantitative tests are completed [120,121] and under regulatory review. More extensive field testing is ongoing to assess the performance of these quantitative G6PD tests in field settings under “real world” conditions and the rates of incorrect test results or interpretations.

The current exclusions to tafenoquine apply to approximately 25% of the population in endemic areas, depending on the G6PD allelic frequency, and fertility and lactation rates [122]. Approving its use in lactating females would substantially improve potential population coverage. Tafenoquine has received regulatory approval in the United States of America, Australia, Brazil, and Thailand [116,117]. Results from the first trial assessing tafenoquine use in children (2 to 15 years) with *P*. *vivax* malaria show that dosing regimens in 4 weight bands were safe and efficacious and reached the target AUC_0-∞_ [60]. As a result, current restrictions on use in children will likely be lifted. Tafenoquine has usually been coadministered with chloroquine. In a recently completed clinical trial in Indonesian soldiers, dihydroartemisinin-piperaquine coadministered with tafenoquine (300 mg dose) was shown to be no different than dihydroartemisinin-piperaquine alone [58] in preventing recurrence of *P*. *vivax* malaria. These data prompted a product label change to Krintafel (the US regulatory approved form of tafenoquine for treatment) in early 2020 [59]. Now, only chloroquine is recommended to be the partner drug to tafenoquine for *P*. *vivax* malaria treatment. If followed this would prevent the use of tafenoquine in areas with chloroquine-resistant *P*. *vivax* parasites where national malaria programmes recommend ACTs for vivax malaria. Clearly, further clinical trials are needed to identify the correct dose of tafenoquine for radical cure [118] and resolve this partner drug uncertainty.

#### How can we identify hypnozoite carriers?

There is currently no test which identifies people with hypnozoites who may later relapse. Living or working in an area of *P*. *vivax* transmission is obviously a risk factor, and having had vivax malaria without radical treatment is clearly the major risk. Pregnant women cannot receive 8-aminoquinolines so are at significant risk if they have vivax malaria during pregnancy. If primaquine could be allowed during lactation (as breast milk excretion is minimal [57]), this should result in a recommendation to give radical cure after delivery. PCR, particularly high-volume uPCR, identifies the majority of asymptomatic *P*. *vivax* infections. Serology can also identify recent infection, although is not widely available.

#### Assessment of therapeutic responses

**Fig 3:**
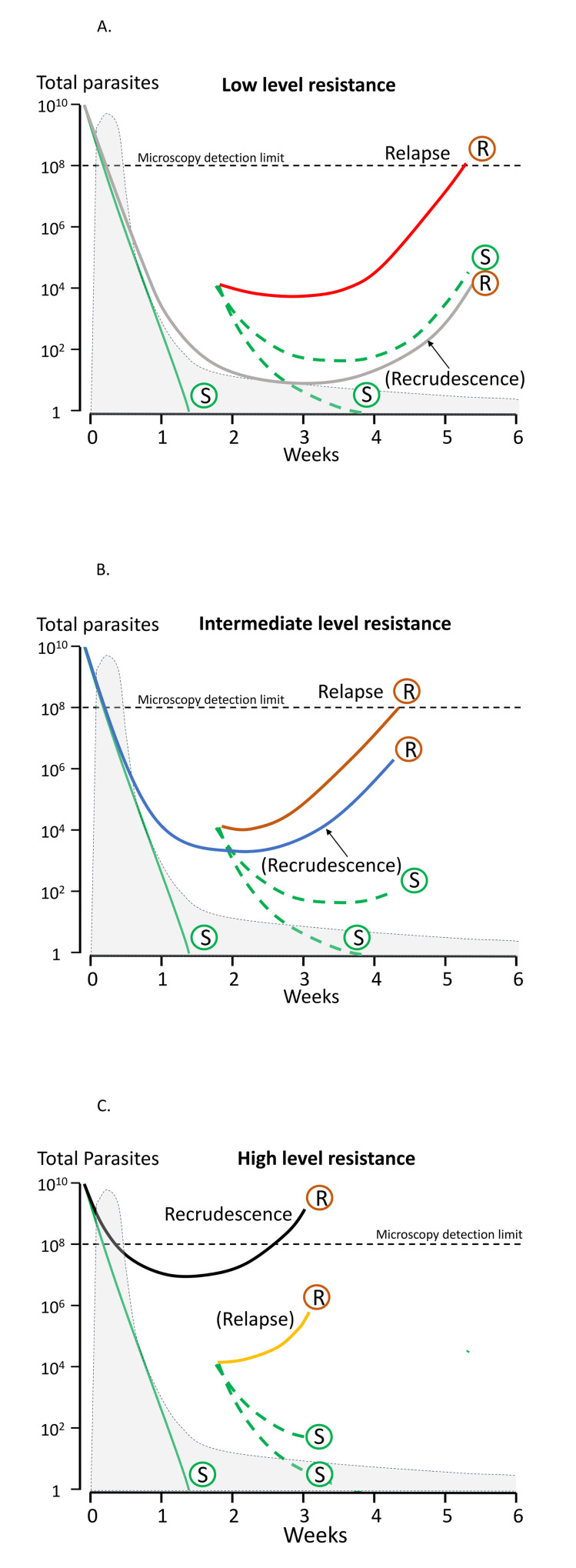
*Plasmodium vivax* chloroquine treatment failure in scenarios of low, intermediate, and high levels of resistance in areas with frequent relapsing *P*. *vivax* [118]. The vertical axis shows the total number of parasites in the body in acute vivax malaria. The light grey shaded area is the blood chloroquine concentration profile. The limit of microscopy detection is approximately 50 parasites/uL. In each panel, chloroquine-sensitive parasites are shown by green lines and marked S. The relapses emerge from the liver approximately 2 weeks after starting treatment. There is uncertainty whether chloroquine eliminates or temporarily suppresses the first relapse (in most cases it suppresses but does not eliminate [118]) so dotted lines representing both scenarios are shown. Resistant parasites causing relapse and recrudescence are marked R. **Upper panel (A):** When there are low levels of resistance, the blood stage infection is cleared usually by the schizonticide, and the first relapse is suppressed until the drug levels fall below the mean inhibitory concentration (MIC) (e.g., in this illustrated example, chloroquine levels above the MIC are maintained until day 28, and relapse parasitaemia becomes patent 2 weeks later) [125]. Recrudescence is very unlikely (occurring only in those patients with low drug levels) and in patients with relapse, the recrudescence would not be detected because the relapse appears first. **Middle panel (B):** When resistance to chloroquine is at an intermediate level, the blood stage infection clears, but the first relapse becomes patent before 28 days. If a relapse occurs, it would still preempt any recrudescence. **Lower panel (C):** With high levels of resistance, the blood stage infection recrudesces before the relapse parasitaemia becomes patent.

As in falciparum malaria, the treatment responses in *P*. *vivax* infections can be assessed by measurement of symptom resolution, fever clearance, and parasite clearance. The lower parasite densities in *P*. *vivax* mean that parasite rate clearance estimates are more difficult to assess, although sequestration is not a confounder in measuring parasite clearance rates because all parasite stages are in the circulation [123]. Quantitative polymerase chain reaction (qPCR) estimation of parasite clearance rates has been used successfully in human challenge experiments but is not widely available [124]. These parasite clearance estimates based on qPCR are not confounded by gametocytaemia (as they are in falciparum malaria) because *P*. *vivax* gametocyte clearance parallels asexual stage clearance after a blood schizonticide [71]. The major challenge in therapeutic assessment is distinguishing relapse, recrudescence, and reinfection, and therefore identifying early resistance. Both chloroquine and the ACT partner drugs are eliminated slowly so that suppressive blood concentrations persist for weeks after treatment [118]. Lumefantrine provides the shortest period of suppression and mefloquine the longest of the currently deployed ACTs. In tropical areas, the first sign of chloroquine resistance in vivo, well before slowing of parasite clearance, is the failure to suppress the first relapse [72,125]. This may not be evident unless patients are followed for 2 months in comparative studies (Fig 3A). As the level of resistance increases, the interval shortens and the first relapse becomes patent within 28 days (Fig 3B). Chloroquine should suppress relapse emergence within 28 days so recurrence within this period, whether from relapse or reinfection, still indicates resistance [125]. If a recurrent infection occurs within 28 days of starting chloroquine for the previous vivax infection, an ACT (such as dihydroartemisinin-piperaquine, artemether-lumefantrine, or artesunate-mefloquine) should be prescribed. Resistance can be confirmed by measuring the whole blood chloroquine level at the time of recurrent parasitaemia [93]. With higher levels of resistance (Fig 3C), recrudescence preempts the relapse. At this stage, parasite genotyping is informative as these early recurrences will all be of the same genotype as the initial infection [29].

## Summary

Frequent recurrent *P*. *vivax* malaria causes substantial preventable morbidity. Better diagnosis, easier G6PD testing, and use of ACTs as an alternative to chloroquine are improving the clinical management of *P*. *vivax* malaria, but the challenge now is to make these more widely available. Primaquine radical cure is under used. Tafenoquine provides single-dose radical cure, but the optimum dose and method of using tafenoquine safely still need to be determined. The tools needed for *P*. *vivax* elimination are available. Malaria elimination activities conducted in remote settings [126–128] have been successful in reducing *P*. *vivax* incidence considerably, but continuing to sustained elimination will require substantial commitment and resources.

## Abbreviations

ACT: artemisinin combination therapy
FST: fluorescent spot test
G6PDd: glucose-6-phosphate dehydrogenase deficiency
IPTi: intermittent presumptive treatment for infants
IPTp: intermittent presumptive treatment for pregnant women
IRS: indoor residual spraying
ITN: insecticide-treated net
LLIN: long-lasting net
PART: presumptive antirelapse therapy
PBO: piperonyl butoxide
PCR: polymerase chain reaction
PvAMA: *P*. *vivax* apical membrane antigen
PvCSP: *P*. *vivax* circumsporozoite protein
PvDBP: *P*. *vivax* Duffy binding protein
PvMSP: *P*. *vivax* merozoite surface protein
qPCR: quantitative polymerase chain reaction
RDT: rapid diagnostic test
SMC: seasonal malaria chemoprevention
uPCR: ultrasensitive PCR
